# Education, Its Influence upon the Eye

**Published:** 1877-10

**Authors:** 


### Editorial.

### Education.

The questions involved in the education of the young in all departments are important ones, and physicians are expected to take a lively interest in them, and be able to advise their friends and the public generally what is truth and what is error. The physician is now being often asked. Does going to school endanger our children's eyes? "Will it make them near-sighted?" If we say yes, or even say there is *danger*, we have pronounced our schools the most intolerable nuisance in the world. If we say no, they present some Ophthalmic Surgeons' report of the number of Myopes in the school, not understanding the object and intention of the report, not understanding that it is for the specialists notoriety rather than the expression of a well settled principle.

That prolonged use of the eye upon small objects, in young children in school *or out of school*, may have influence upon the form of the globe, and in some cases produce Myopia seem probable, but that school-desks, school-light or other conditions of school-life, produce deviations in the form of the globe not found elsewhere, is in our opinion highly improbable.

That one child should have myopia, and another hypermetropia, conditions exactly the reverse of each other from the same cause, cannot be accepted as philosophical. It is certain that young students are often myopic, born myopic, they are so before attending school, and often in still greater degree after they have completed student life, that the same conditions are to be found in those who have never attended school--whose early education has been obtained out of school, or even neglected altogether. If there is manifest defect in school light, desk or other condition, it will be found easier to remedy it than to correct the abnormal refraction of the eye or the evil and inconvenience of ignorance, and general neglect of school-life. In directing the course of study, many mistakes are constantly made. If the discovery that our school-children are near-sighted in alarming frequency, shows anything at all, it would seem to point to a general truth pretty well understood, that children are too early sent to the school-room, and too closely confined to it. Public sentiment and law should prevent parents from gratifying their pride and foolishness by sending children to school before they are of proper age. Commendable efforts are already inaugurated in some cities, and states, for preventing this cruelty to children, and it is greatly to be hoped it will soon become universal.

We are ready to admit, that too early and too prolonged use of the eye upon minute objects, fine type for instance, is adequate cause of myopia, but we cannot see how it should in some cases produce lengthening of the globe of the eye, and in others shortening. It is however, difficult to determine what the influence of school-life is upon school children, and to distinguish those effects which may be fairly attributed to life inside the school-room from those which are reasonably to be assigned to conditions outside the school-room.

Cohen many years ago, published very instructive reports of the condition of the eyes of the children, both in Breslau and the surrounding villages, showing that children in the village schools had myopia early, in not more than two per cent. and this he regarded as the normal condition. Schools as they advanced in grade afforded a progressive near-sightedness among the pupils until the University was reached, when he found a fearful frequency, one half being myopic.

Others have imitated Cohen, of Breslau, and American cities and villages have been brought under tribute for statistics, until now we are believed by the public to have such light, or desks, or type in our schools as to cause near-sightedness in alarming frequency, Cohen, in Germany, and distinguished Ophthalmologists everywhere pointing out our schools as causing near-sightedness, and at the same time pointing out their own accurate knowedge of the laws of refraction and accommodation to all thoughtful parents through the medium of the children of the common schools. The influence of school life, if injurious to our children, is most certainly to be pointed out, since no wise or humane parent would continue his child in school, when once informed and made to believe that it in any way endangered the organ of vision. Convince the public of this, and common schools will be no longer popular or even tolerable.

